# Podocalyxin, Isthmin-1, and Pentraxin-3 Immunoreactivities as Emerging Immunohistochemical Markers of Fibrosis in Chronic Hepatitis B

**DOI:** 10.3390/biomedicines13122958

**Published:** 2025-12-01

**Authors:** Müge Özgüler, Serhat Hançer, Özgen Arslan Solmaz, Tuncay Kuloğlu

**Affiliations:** 1 Department of Infectious Diseases and Clinical Microbiology, Elazığ Fethi Sekin City Training, and Research Hospital, University of Health Sciences, 23280 Elazıg, Turkey; 2 Department of Histology and Embriology, Ministry of Health, Gaziantep City Hospital, 27060 Gaziantep, Turkey; 3 Department of Pathology, Elazığ Fethi Sekin City Training, and Research Hospital, University of Health Sciences, 23280 Elazıg, Turkey; 4 Department of Histology and Embriology, Medical Faculty, Fırat University, 23119 Elazıg, Turkey

**Keywords:** chronic hepatitis B, liver fibrosis, Podocalyxin, Pentraxin-3, Isthmin-1

## Abstract

**Introduction and Objectives:** Persistent hepatic inflammation serves as a key driver of fibrogenesis in chronic hepatitis B. Fibrosis is a complex molecular and cellular process. Podocalyxin is a type I transmembrane sialomucin, and physiological expression of podocalyxin has been identified in the liver. Pentraxin 3 plays a crucial role in humoral innate immune responses. Isthmin-1 has been associated with metabolic regulation and immune response modulation. We aimed to evaluate the immunoreactivities of podocalyxin, Isthmin-1, and pentraxin-3 in the liver tissue of patients with chronic hepatitis B. **Materials and Methods:** Power analysis was performed (effect size (f = 0.5), (α) = 0.05 and statistical power of 0.80). Sample size was calculated to be a total of 63 samples, with 21 samples per group. Individuals with negative hepatitis serology and normal liver histopathology, from whom liver tissue was obtained for any reason, were designated as the control group. Liver specimens of chronic hepatitis B were categorized into F0–F2 (no or mild fibrosis) and ≥F3 (advanced fibrosis). Immunohistochemical staining was performed to assess the expression and immunoreactivity of Podocalyxin, Isthmin-1, and Pentraxin-3. A histoscore was created based on the prevalence of staining immunoreactivity (0.1: <25%, 0.4: 26–50%, 0.6: 51–75%, 0.9: 76–100%) and intensity (0: none, +0.5: very low, +1: low, +2: moderate, +3: severe). **Results:** A statistically significant increase in Podocalyxin, Pentraxin-3, and Isthmin-1 immunoreactivities was found in fibrotic liver tissue compared to normal liver tissue and mild fibrotic groups (*p* < 0.05). **Conclusions:** We concluded that our findings suggest these proteins may have an additional role in the progression of liver fibrosis in chronic hepatitis B.

## 1. Introduction

Based on data from the Global Hepatitis Report 2024, it was estimated that in 2022, around 254 million individuals globally were chronically infected with hepatitis B virus (HBV). Without appropriate medical intervention, chronic HBV infection poses a serious risk of progressive liver disease, including hepatic fibrosis, cirrhosis, and the development of hepatocellular carcinoma [1].

Hepatic fibrogenesis represents a complex interplay of molecular and cellular mechanisms, primarily driven by activated, migratory, and profibrogenic cell populations. This pathological process is characterized by the progressive deposition of extracellular matrix (ECM) components within the liver parenchyma, leading to the transformation of normal hepatic tissue into fibrotic tissue [2,3]. Moreover, aberrant angiogenesis is increasingly recognized as a contributing factor in liver fibrosis progression, as it facilitates the persistence and expansion of fibrotic lesions [4].

Cirrhosis entails extensive structural reorganization of the liver, characterized by the development of regenerative nodules surrounded by fibrous septa and notable alterations in hepatic vascular integrity [5]. Patients exhibiting with progressive liver fibrosis face a significantly elevated risk of developing hepatocellular carcinoma (HCC) [6].

In the context of chronic hepatitis B, persistent hepatic inflammation serves as a key driver of fibrogenesis. This chronic inflammatory state, maintained by ongoing hepatocellular injury, facilitates the recruitment and activation of immune cells, which in turn initiate the differentiation of profibrogenic cells into hepatic myofibroblasts (MFs). This transformation is mediated by a complex network of signaling molecules, including cytokines, chemokines, and reactive oxygen species (ROS). Once activated, MFs promote a profibrotic microenvironment through excessive deposition of extracellular matrix components, sustained production of inflammatory mediators, and interacting with immune cells. These alterations interfere with normal hepatocyte proliferation and tissue regeneration in the liver parenchyma [7]. A pivotal event in this cascade is the transdifferentiation of hepatic stellate cells (HSCs), which results in the substitution of collagen type IV with fibrillar collagens types I and III, contributing to the capillarization of hepatic sinusoids. Concurrently, the accumulation of fibrous septa advances the liver toward cirrhosis, accompanied by both hypoxia-driven and independent pathological angiogenic processes [8].

Podocalyxin (PODXL), a member of the CD34 family, is a type I transmembrane sialomucin predominantly localized to the apical membrane of cells, with high expression notably observed in the glomerular epithelium of the kidney [9]. Beyond the renal system, PODXL has been detected on various cell types, including vascular endothelial cells, megakaryocytes, platelets, mesothelial cells, hematopoietic progenitors, and certain neurons. Within epithelial tissues, PODXL is implicated in key biological functions such as embryogenesis, immune modulation, and the metastatic progression of cancer. These roles are facilitated through its interactions with adaptor proteins linked to actin binding, protein transport, and signal cascades [10]. Physiological expression of PODXL has been identified in organs such as the kidney, liver, pancreas, breast, and endometrium. Functionally, it contributes to the regulation of cell adhesion and is associated with the acquisition of aggressive traits in epithelial malignancies, promoting enhanced tumor cell proliferation, invasion, motility, and metastatic capacity. Accordingly, elevated PODXL expression in the context of advanced tissue fibrosis has been proposed as a potential biomarker for malignant transformation [10,11].

Pentraxin 3 (PTX3), also referred to as tumor necrosis factor-stimulated gene 14 (TSG-14), is a member of the long pentraxin family within the pentraxin superfamily. It plays a crucial role in humoral innate immune responses, particularly in the early defense against pathogens, and is recognized for its involvement in the interplay between inflammation, tissue remodeling, and neoplastic transformation [12]. In the context of hepatic disorders, circulating PTX3 has been implicated in the pathophysiology of non-alcoholic steatohepatitis (NASH) [13]. Elevated PTX3 concentrations have also been observed in cases of acute liver injury induced by paracetamol overdose [14]. Clinically, serum PTX3 has shown promise as a non-invasive biomarker for assessing liver fibrosis in patients with chronic hepatitis C virus (HCV) infection [15]. Moreover, elevated plasma levels of PTX3 have been linked to an increased risk of hepatocellular carcinoma (HCC) development in individuals with chronic HCV infection [16].

Isthmin-1 (ISM1) has been associated with a range of biological and pathological processes, including metabolic regulation, immune response modulation, tumor development, cellular proliferation, and the control of endothelial barrier permeability. Expression of ISM1 is predominantly observed in neural and respiratory tissues such as the brain, lungs, bronchi, alveolar epithelium, cerebellum, cerebral cortex, and hippocampus [17]. In addition, variable expression levels have been reported in organs such as eye, kidneys, skeletal muscle, and myocardium. ISM1 is known to influence lymphocyte activity and is suggested to participate in both innate and adaptive immune responses [18]. Emerging evidence points to a potential role for ISM1 in liver fibrosis, particularly through its involvement in the activation of hepatic stellate cells (HSCs), which are central mediators of fibrogenesis. It is hypothesized that ISM1 may coordinate intercellular communication during hepatic injury by interacting with immune mediators such as cytokines and growth factors. Elevated ISM1 expression may serve as an indicator of fibrotic burden and disease progression in chronic hepatitis B. However, to date, studies exploring the role of ISM1 in chronic hepatitis B are lacking; current literature is largely restricted to its involvement in hepatic steatosis.

The present study aimed to evaluate the immunohistochemical expression of podocalyxin, isthmin-1, and pentraxin-3 in liver biopsy specimens from patients with chronic hepatitis B, and to compare their expression levels between individuals with early-stage (no or mild) fibrosis and those with advanced fibrosis.

## 2. Materials and Methods

### 2.1. Study Design

This study was designed as a retrospective analysis. It was conducted in accordance with the principles of the Declaration of Helsinki and approved by the Non-Interventional Research Ethics Committee of Elazığ Fethi Sekin Training and Research Hospital (Protocol No: 2025/8-7; Date of approval: 24 April 2025). The sample size was determined based on the number of available liver tissue preparations archived in the pathology department from patients diagnosed with chronic hepatitis B. Liver biopsy specimens were obtained from patients who were evaluated at the Department of Infectious Diseases and Clinical Microbiology of Elazığ Fethi Sekin City Hospital between August 2018 and July 2024, and who were diagnosed with chronic hepatitis B based on histopathological findings. Individuals with negative hepatitis serology and normal liver histopathology, from whom liver tissue was obtained for any reason were designated as the control group. Liver tissue samples were categorized into two groups based on fibrosis stage: F0–F2 (no-mild fibrosis) and ≥F3 (advanced fibrosis). Immunohistochemical staining was performed to assess the expression and immunoreactivity of Podocalyxin, Isthmin-1, and Pentraxin-3 in the liver tissues.

### 2.2. Inclusion Criteria

- Participants were included in the study if they met all of the following conditions:
- Aged 18 years or older;
- Underwent liver biopsy for the diagnosis of chronic hepatitis B, with pretreatment histopathological evaluation of fibrosis by the pathology department;
- No co-infections known to affect liver fibrosis, such as hepatitis C virus (HCV) or hepatitis D virus (HDV);
- No evidence of acute hepatitis at the time of liver biopsy;
- No diagnosis of acute exacerbation of chronic hepatitis at the time of liver biopsy;
- Absence of any concurrent malignancy;
- Not receiving immunosuppressive therapy;
- No co-infection with human immunodeficiency virus (HIV);
- Not on medication for any chronic systemic illness (e.g., hypertension, diabetes mellitus);
- No additional condition that could contribute to chronic hepatic ischemia;
- No history of direct surgical intervention involving the liver.

### 2.3. Exclusion Criteria

Participants were excluded from the study if they met any of the following conditions:

- Younger than 18 years of age;
- Did not undergo liver biopsy for the diagnosis of chronic hepatitis B;
- Presence of co-infection affecting liver fibrosis (e.g., HCV or HDV);
- Diagnosis of acute hepatitis at the time of liver biopsy;
- Diagnosis of acute exacerbation of chronic hepatitis at the time of liver biopsy;
- Presence of concurrent malignancy;
- Currently receiving immunosuppressive therapy;
- Co-infection with HIV and undergoing treatment for it;
- Receiving medication for any chronic systemic illness (e.g., hypertension, diabetes mellitus);
- Presence of comorbidities that may contribute to chronic hepatic ischemia (e.g., congestive heart failure, cardiac arrhythmias, arterial ischemia);
- History of direct surgical intervention involving the liver.

### 2.4. Evaluation of Liver Fibrosis

Histopathological evaluation of liver biopsy specimens was performed prior to the initiation of hepatitis B treatment. The staging and grading of liver histology were conducted according to the modified Ishak fibrosis staging score. The Ishak score assesses the extent of liver fibrosis on a scale ranging from F0 to F6, according to the following definitions [19]:

- F0: No fibrosis;
- F1: Fibrous expansion in some portal areas;
- F2: Fibrous expansion in most portal areas;
- F3: Fibrous expansion in most portal areas with occasional portal–portal bridging;
- F4: Marked portal–portal and portal–central bridging;
- F5: Extensive bridging with occasional nodules (incomplete cirrhosis);
- F6: Cirrhosis.

For the purposes of this study, fibrosis stages were grouped as follows: no or mild fibrosis ≤ F2 and significant, advanced fibrosis and cirrhosis ≥ F3.

### 2.5. Immunohistochemistry

Sections 4–6 µm thick were taken from paraffin blocks and placed on polylysine-coated slides. The deparaffinized tissues were passed through graded alcohol series and boiled in a microwave oven (750 W) for 15 min in citrate buffer solution at pH 6 for antigen retrieval. After boiling, the tissues were allowed to cool at room temperature for approximately 20 min, were washed with PBS (Phosphate-Buffered Saline, P4417, Sigma-Aldrich, St. Louis, MO, USA) 3 times for 5 min each, then incubated for 5 min with hydrogen peroxide blocking solution (Hydrogen Peroxide Block, TA-125-HP, Lab Vision Corporation, Fremont, CA, USA) to inhibit endogenous peroxidase activity. After washing the tissues three times for 5 min each with PBS, 5 min of Ultra V Block (TA–125-UB, Lab Vision Corporation) solution was applied to prevent background staining, followed by incubation with PTX3, PODXL, and ISM-1 primary antibodies diluted at a ratio of 1/200 (PTX3 Polyclonal Antibody, PA5-36156, Thermo Fisher Scientific, Invitrogen, Waltham, MA, USA/PODXL, 39-3800, Thermo Fisher Scientific Inc./ISM1 Polyclonal Antibody, E-AB-18133, Elabscience Biotechnology, Houston, TX, USA) for 60 min at room temperature in a humidified environment.

After washing the tissues with PBS 3 times for 5 min each following the application of the primary antibody, they were incubated with the secondary antibody (biotinylated Goat Anti-Polyvalent (anti-mouse/rabbit IgG), TP–125-BN, Lab Vision Corporation) for 30 min at room temperature in a humid environment. The tissues were washed 3 times for 5 min each with PBS after the secondary antibody application, then incubated with Streptavidin Peroxidase (TS–125-HR, Lab Vision Corporation) for 30 min at room temperature in a humid environment, and finally placed back into PBS. After applying 3-amino-9-ethylcarbazole (AEC) Substrate + AEC Chromogen (AEC Substrate, TA-015 and HAS, AEC Chromogen, TA-002-HAC, Lab Vision Corporation) solution onto the tissues and obtaining the image signal under the light microscope, the tissues were simultaneously washed with PBS. Tissues stained with Mayer’s hematoxylin in a contrasting manner were rinsed with PBS and distilled water, then mounted with an appropriate mounting medium (Large Volume Vision Mount, TA-125-UG, Lab Vision Corporation). The prepared slides were examined and evaluated under a Leica DM500 microscope (Leica Microsystems, Wetzlar, Germany) and photographed (Leica DFC295, Leica Microsystems, Wetzlar, Germany).

### 2.6. Evaluation of Immunoreactivity

A histoscore was created based on the prevalence of staining immunoreactivity (0.1: <25%, 0.4: 26–50%, 0.6: 51–75%, 0.9: 76–100%) and intensity (0: none, +0.5: very low, +1: low, +2: moderate, +3: severe). Histoscore = prevalence x intensity. Two histologists and one pathologist evaluated the slides independently.

### 2.7. Statistical Analysis

The data obtained were expressed as mean ± standard deviation. The SPSS version 22 software was used for statistical analysis. The Shapiro–Wilk test was used for the normality analysis. Intergroup comparisons were performed using One-way ANOVA and Post hoc Tukey tests. Values of *p* < 0.05 were considered statistically significant. Sample size estimation was conducted using G*Power 3.1 software program. Power analysis was performed to determine the minimum number of sample required to detect a statistically significant difference among three independent groups (low-grade fibrosis, high-grade fibrosis and control) using a one-way ANOVA. The analysis assumed a large effect size (f = 0.5), a significance level (α) of 0.05 and statistical power of 0.80. Based on these parameters, the required sample size was calculated to be total of 63 samples, 21 samples per group.

## 3. Results

A total of 63 liver tissue specimens were included in the study. According to the ISHAK fibrosis staging system, liver tissues with a stage of ≤2 were classified as low-grade fibrosis, while those with a stage of ≥3 were classified as high-grade fibrosis. The main characteristics of the patients according to fibrosis groups are presented in Table 1.

**Figure 1:**
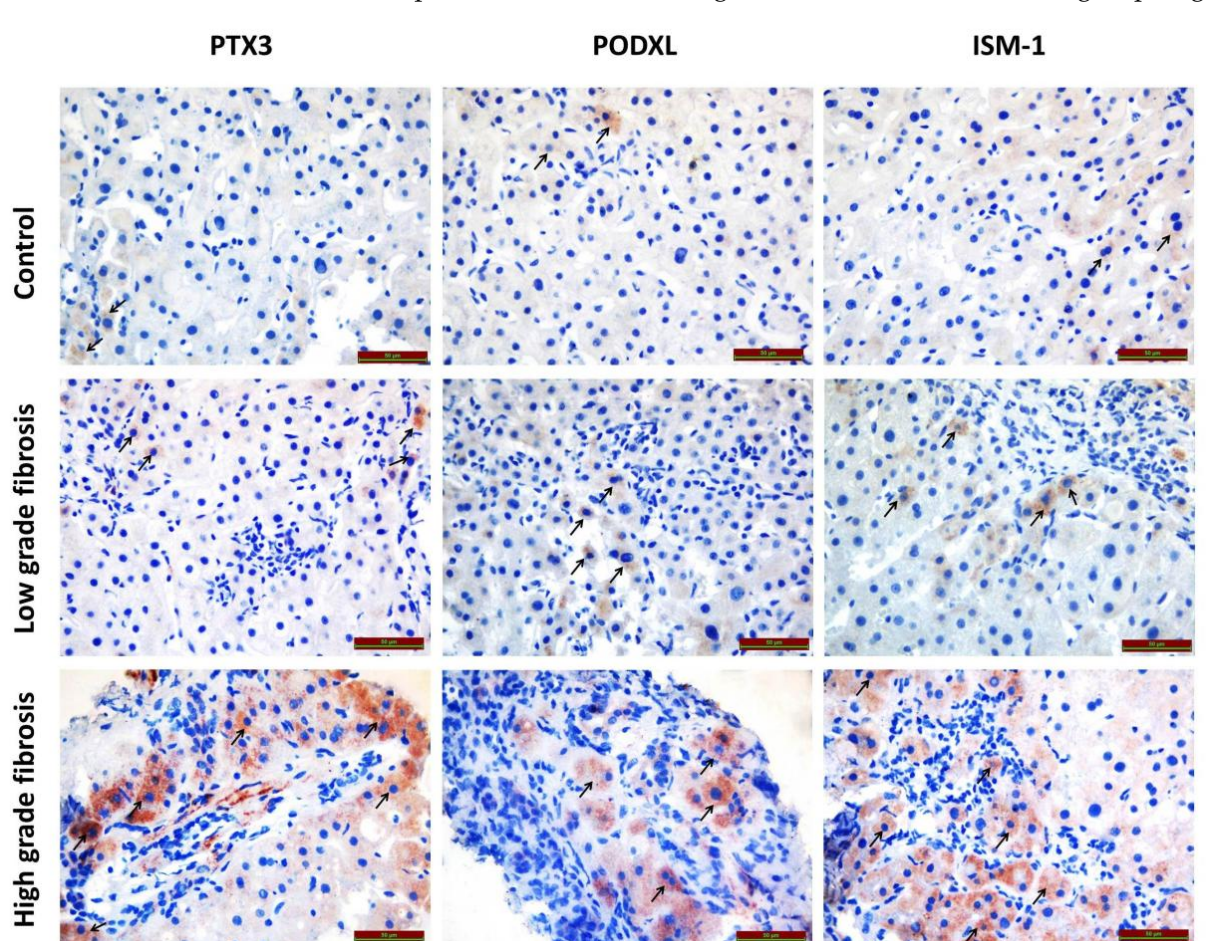
The immunoreactivities of PODXL, PTX3 and ISM-1 in the low fibrosis, high fibrosis, and control groups. (Arrows indicate immunoreactivities).

In our study, the immunoreactivities of Podocalyxin, Isthmin-1, and Pentraxin-3 in the liver tissues of patients with chronic hepatitis B and high-grade fibrosis were found to be increased compared to those with low-grade fibrosis and the control group (Figure 1).

### 3.1. Immunohistochemical Findings

In the evaluation of histoscores obtained from immunohistochemical staining for PODXL, PTX3, and ISM1, a statistically significant difference was observed between liver tissues with fibrosis associated with chronic hepatitis B and those from the control group. Furthermore, the immunoreactivity levels of PODXL, PTX3, and ISM1 were found to increase significantly in parallel with the progression of fibrosis. The histoscore-based immunoreactivity results for PODXL, PTX3, and ISM1 are summarized in Table 2.

#### 3.1.1. PODXL Immunoreactivity

As a result of examining the immunohistochemical staining performed to determine PODXL immunoreactivity under a light microscope; compared to the control group, a statistically significant increase in PODXL immunoreactivity was found in the low-grade fibrosis (*p* = 0.001) and high-grade fibrosis (*p* < 0.001) groups. Furthermore, compared to the low-grade fibrosis group, a statistically significant increase in PODXL immunoreactivity was observed in the high-grade fibrosis group (*p* = 0.004).

#### 3.1.2. PTX3 Immunoreactivity

As a result of examining the immunohistochemical staining performed to determine PTX3 immunoreactivity under a light microscope; compared to the control group, a statistically significant increase in PTX3 immunoreactivity was detected in the low-grade fibrosis (*p* = 0.003) and high-grade fibrosis (*p* < 0.001) groups. Furthermore, compared to the low-grade fibrosis group, a statistically significant increase in PTX3 immunoreactivity was observed in the high-grade fibrosis group (*p* = 0.017).

#### 3.1.3. ISM1 Immunoreactivity

The examination of ISM1 immunoreactivity, determined by immunohistochemical staining and evaluated under a light microscope, compared to the control group, a statistically significant increase in ISM1 immunoreactivity was found in both the low-grade fibrosis (*p* = 0.036) and high-grade fibrosis (*p* < 0.001) groups. Furthermore, when compared to the low-grade fibrosis group, a statistically significant increase in ISM1 immunoreactivity was observed in the high-grade fibrosis group (*p* = 0.016).

The immunoreactivities of PODXL, PTX3 and ISM-1 in the low fibrosis, high fibrosis, and control groups are shown in Figure 1.

## 4. Discussion

In this study, PODXL, PTX3, and ISM1 demonstrated immunoreactivity in fibrotic liver tissue when compared to normal liver tissue. Additionally, the histoscore reflecting immunoreactivity was found to increase statistically significantly with the advancing degree of fibrosis. This correlation does not exclude the possibility that these three proteins may promote the progression of liver fibrosis. Despite these findings, there is a scarcity of research in the literature regarding these three markers, and the majority of existing studies have focused on their serum levels.

Angiogenesis has been recognized as a key mechanism in the progression of liver fibrosis, playing a critical role in supporting disease development and enabling tissue remodeling [20]. The formation of new blood vessels enhances the survival and activation of fibrogenic cells by improving the supply of oxygen and nutrients. Simultaneously, neovascularization facilitates the infiltration of inflammatory cells and the release of cytokines, both of which further drive fibrotic progression [21]. Moreover, hypoxia is a potent stimulus for angiogenesis, suggesting that oxygen deficiency in fibrotic regions contributes to the expansion of the new blood vessels [22].

PODXL has been identified on the surface of vascular endothelial cells, indicating its significant involvement not only in vascular function but also in epithelial cell physiology [23]. Previous studies have highlighted that PODXL overexpression correlates with poor prognosis and adverse clinical outcomes in various epithelial malignancies [23,24,25,26]. Additionally, growing evidence supports the involvement of PODXL and CD34 in neovascularization during embryonic development and solid tumor progression, as well as in preserving vascular integrity in inflamed tissues [27].

In normal liver tissue, PODXL expression is confined to hepatic arterioles. Additionally, PODXL has been detected on the sinusoidal endothelial cells of hyperplastic focal nodules and hepatic adenomas [28]. However, to date, the immunoreactivity of PODXL in liver tissue of individuals with chronic hepatitis B has not been systematically investigated. Its expression may serve as an additional diagnostic marker in the histopathological evaluation of liver biopsies.

The majority of studies on PODXL have focused on its role in hepatocellular carcinoma (HCC). High levels of PODXL expression have been reported in sinusoidal endothelial cells and tumour-like hepatic lesions, whereas adjacent non-tumorous liver tissues typically show little or no expression [28,29,30]. In prior research, PODXL1 expression was assessed via immunohistochemical staining in liver tissue. It was found to be present in tumour-associated microvascular endothelial cells in HCC, as well as in the capillarized sinusoidal endothelium of focal nodular hyperplasia (FNH) and hepatic adenomas [30]. In cirrhotic nodules, PODXL1 expression showed a correlation with CD34 and prominently marked the endothelial cells in the inflow regions. In contrast, in dysplastic nodules, expression of both CD34 and PODXL1 was either absent or only focal. These findings suggest that PODXL holds diagnostic value in distinguishing HCC from cirrhotic lesions in liver biopsies [28]. Furthermore, another study proposed PODXL as a potential biomarker for the diagnosis of liver cancer [29]. In our study, we observed significant PODXL immunoreactivity in liver tissue of patients with chronic hepatitis B. Importantly, we found a progressive increase in expression levels from low-grade to high-grade fibrosis. These findings are consistent with existing literature, particularly regarding the relationship between fibrosis progression and increased angiogenesis.

Based on our current knowledge, this is the first study in the literature to directly investigate the immunoreactivity of PTX3 in liver tissue in CHB. While several previous studies have evaluated serum PTX3 levels in the context of liver fibrosis and hepatocellular carcinoma (HCC), tissue-level expression has not been evaluated. PTX3 has been implicated in the pathogenesis of hepatic inflammation and fibrogenesis [31].

PTX3 expression can be induced by pro-inflammatory cytokines such as tumor necrosis factor (TNF)-α and interleukin (IL)-1 [32]. In particular, IL-1β levels have been shown to increase progressively with the advancement of chronic hepatitis B virus (HBV) infection toward HCC [33]. Furthermore, IL-10 has been reported to promote PTX3 production by stimulating B cells as part of the adaptive immune response [34]. Elevated IL-10 levels have been associated with disease progression from the inactive carrier state to cirrhosis and eventually to HCC in HBV-infected individuals [35]. Although hepatocytes are not considered a primary source of PTX3 [36], studies have demonstrated that PTX3 can promote the proliferation of HCC cells and induce epithelial–mesenchymal transition a process closely linked to tumor cell invasion and metastasis [37].

In a previous study, a total of 516 participants were enrolled, including 365 patients with chronic hepatitis B virus (CHB) infection (comprising 159 with chronic hepatitis, 99 with cirrhosis, and 107 with HBV-related HCC, as well as 151 healthy controls. The study revealed that serum PTX3 levels were significantly higher in patients with chronic HBV infection compared to healthy individuals, and notably elevated in those with HBV-related HCC compared to patients with chronic hepatitis or cirrhosis. Furthermore, elevated serum PTX3 was identified as an independent risk factor for the development of HCC [12]. Notably, serum PTX3 demonstrated strong discriminatory power in distinguishing HCC from chronic hepatitis, cirrhosis, and chronic HBV infection without HCC. This discriminative capacity was not affected by the presence of underlying liver disease (chronic hepatitis or cirrhosis) or by the administration of antiviral therapy.

Importantly, PTX3 levels were especially effective in identifying α-fetoprotein (AFP)-negative and early-stage HCC, distinguishing it from chronic hepatitis, cirrhosis, and chronic HBV infection without HCC. These findings support the hypothesis that PTX3 contributes to the progression of HBV-related liver disease and the development of HCC. As such, PTX3 may serve as a valuable diagnostic biomarker for HBV-associated HCC, particularly in cases where AFP levels are negative or the disease is at an early stage [12]. In addition, elevated plasma PTX3 levels have been identified as a risk factor for HCC development in patients with chronic hepatitis C virus (HCV) infection [16]. Increased PTX3 expression in tumor tissues has also been associated with poorer prognosis in individuals with HCC [37].

In a separate study, PTX3 levels were found to be significantly higher in patients with non-alcoholic steatohepatitis (NASH) compared to those with simple steatosis [13]. Additionally, PTX3 levels have been shown to correlate with the severity of liver fibrosis in both NASH [38] and HCV infection [15]. These findings highlight the potential of PTX3 as a more sensitive biomarker for HCC, particularly for early-stage disease. In our study, we observed a significant increase in PTX3 immunoreactivity in liver tissue in advanced stages of liver fibrosis of CHB. In this study, we exclusively evaluated PTX3 immunoreactivity in liver tissue; however, we were not able to assess plasma PTX3 levels at the same time. However, the increased PTX3 expression observed in liver tissue in our study suggests that our findings are in line with previous studies reporting elevated plasma PTX3 levels, thereby supporting their conclusions.

To date, there are no studies investigating ISM-1 immunoreactivity in fibrotic liver tissue associated with CHB. ISM-1, initially identified as an insulin-like adipokine, has been shown to enhance glucose uptake in adipocytes and muscle cells, while inhibiting hepatic lipid synthesis. It also suppresses lipogenesis and promotes protein synthesis in hepatocytes through insulin-independent mechanisms [39,40]. Despite these findings, the precise biological role of ISM-1 remains largely unclear. In adulthood, ISM-1 is expressed in various tissues and organs in an organ- and tissue-specific manner [17].

Current research on the biological functions of ISM-1 primarily focuses on its roles in growth and development, metabolism, and cancer treatment. ISM-1 affects cellular processes such as autophagy, angiogenesis, and the immune microenvironment, particularly in cancer progression [41]. ISM-1 plays a significant role in promoting apoptosis and anti-angiogenesis, while regulating multiple inflammatory pathways that influence the immune response [42,43].

Studies have confirmed that both ISM-1 and Fibroblast Growth Factor 8 (FGF-8) (previously identified as a critical signaling molecule) are expressed in similar regions, exhibiting partially overlapping or co-expressed domains [44,45,46]. These findings suggest that ISM-1 may play a role in the pathogenesis of fibrosis in chronic hepatitis B (CHB).

ISM1 is overexpressed in Th17 cells, whereas its expression is low in regulatory T cells (Tregs) and absent in Th1 and Th2 subsets. This expression pattern suggests that ISM1 may contribute to the biological activities of NK, NKT, Th17, and other immune cells, thereby playing a role in immune regulation and related processes [18].

Wu et al. [47] demonstrated through gene enrichment analysis that ISM1 is strongly associated with key immune signaling pathways, including TGF-β, IL-6/JAK/STAT3, IFN-γ, TNF-α/NF-κB, and IL-2/STAT5. These pathways are known to influence Treg cell infiltration, the stability of programmed death-ligand 1 (PD-L1), and CD8+ T cell exhaustion—all of which are relevant to the development of liver fibrosis.

Additionally, ISM1 expression correlates with markers of T-cell exhaustion further supporting the hypothesis that ISM1 may suppress the immune response [47]. These findings point to a dual role for ISM1 in immune regulation: it may suppress immune activity via classical pro-inflammatory pathways, while simultaneously enhancing immune responses through type I interferon signaling. This complex immunomodulatory role of ISM1 remains to be fully elucidated [41]. In our study, we observed increased ISM1 immunoreactivity in liver tissues with moderate to advanced fibrosis induced by chronic hepatitis B (CHB). These findings support the potential of ISM1 as a novel marker in the progression of liver fibrosis.

In conclusion, in our study, we observed that the immunoreactivities of PODXL, PTX3, and ISM1 in liver tissue were increased in fibrotic livers associated with chronic hepatitis B, compared to liver tissues with normal or low-grade fibrosis. These findings suggest that these proteins may have an additional role in the progression of liver fibrosis. We believe that our results may provide a basis for future, large-scale studies in this area, and that the data obtained could contribute to the development of novel diagnostic tools and therapeutic strategies.

## 5. Limitation of Study

We recognized some limitations of the study. First, this is a retrospective study in a relatively small number of patients with chronic HBV infection. Nevertheless, this study demonstrated consistent findings for the diagnostic contribution in immunreactivation of PODXL, PTX3, and ISM-1 in the liver tissue of CHB-infected individuals. We believe that further studies with larger sample sizes examining the correlation with serum levels would contribute to the literature on this subject.

## Figures and Tables

**Table 1:** Main characteristics of patients according to fibrosis group.

Characteristics	Low-Grade Fibrosis (*n* = 21) (Mean ± STD)	High-Grade Fibrosis (*n* = 21) (Mean ± STD)	Total (Mean ± STD)
Age (Mean)	36.7 ± 10.2	47.5 ± 15.4	42.1 ± 14.0
Sex (F/M)	8/13	4/17	12/30
AST (U/L)	27.4 ± 9.4	63.0 ± 51.4	45.2 ± 41.0
ALT (U/L)	35.4 ± 26.0	91.8 ± 93.9	63.6 ± 74.5
Total protein (g/L)	71.5 ± 3.3	71.5 ± 5.8	72.0 ± 4.7
Albumin (g/L)	42.8 ± 2.1	41.5 ± 4.7	42.1 ± 3.7
INR	1.03 ± 0.07	1.05 ± 0.09	1.04 ± 0.08
ALP (U/L)	82.5 ± 29.3	86.1 ± 23.8	84.3 ± 26.8
GGT (U/L)	16.0± 4.9	24.9 ± 16.3	20.7 ± 12.7
Platelet count (10^9^/L)	239 ± 37	194 ± 42.9	216 ± 45
HBV DNA IU/mL	200,265 ± 702,864	76,300,956 ± 120,670,886	38,250,611 ± 93,428,085
HBeAg positive/negative	1/20	6/15	7/35

AST: Aspartate Aminotransferase, ALT: Alanine Aminotransferase, ALP: Alkaline Phosphatase, GGT: Gamma-Glutamyl Transferase, INR: International Normalized Ratio, HBV DNA: Hepatitis B Virus Deoxyribonucleic Acid, HBeAg: Hepatitis B e Antigen, STD: Standard Deviation.

**Table 2:** Immunoreactivity of PODXL, ISM1 and PTX3 according to Histoscore.

Immunoreactivity Histoscore	Control (n = 21)	Low-Grade Fibrosis (n = 21)	High-Grade Fibrosis (n = 21)	*p* Value *
PODXL	0.29 ± 0.10	0.78 ± 0.21 ^a^	1.20 ± 0.63 ^b^	<0.001
PTX3	0.40 ± 0.08	0.74 ± 0.19 ^a^	1.01 ± 0.48 ^b^	<0.001
ISM1	0.46 ± 0.07	0.88 ± 0.47 ^a^	1.35 ± 0.75 ^b^	<0.001

Values are presented as mean ± standard deviation. * One way ANOVA test, ^a^ Compared to the Control group, ^b^ Compared to the low-grade fibrosis group (*p* < 0.05).

## Data Availability

The original contributions presented in this study are included in the article. Further inquiries can be directed to the corresponding author.
